# Is Dextrose Prolotherapy More Effective Than Steroids in the Treatment of Distal Semimembranous Tendinopathy?

**DOI:** 10.7759/cureus.70663

**Published:** 2024-10-01

**Authors:** Emine Yıldırım Uslu

**Affiliations:** 1 Physical Medicine and Rehabilitation, Elazığ Fethi Sekin City Hospital, Elazığ, TUR

**Keywords:** dextrose prolotherapy, distal semimembranous tendinopathy, steroid injection, tendinopathy, ultrasound guided injection

## Abstract

Objective: Distal semimembranous (SM) tendinopathy is one of the rare causes of posteromedial knee pain. Steroid injections are included in the treatment of resistant cases. The effectiveness of dextrose prolotherapy is not fully known. This study aimed to investigate the effectiveness of dextrose prolotherapy in distal SM tendinopathy and to compare it with the effectiveness of steroid injections.

Methodology: The study included 12 patients who received ultrasound-guided steroid injections and 13 patients who received dextrose prolotherapy treatment with the diagnosis of distal SM tendinopathy. The patient's visual analog scale (VAS) and International Knee Documentation Committee Subjective Knee Assessment (IKDC) scores were analyzed before the procedure, as well as one week and four weeks afterward.

Results: Pre-procedure VAS scores were similar in the dextrose and steroid groups (4.00 ± 0.70, 4.33 ± 0.88 *P *= 0.308), while they were significantly lower in the steroid group at week 1 (1.84 ± 0.55, 1.16 ± 0.93, *P *= 0.036). Fourth-week VAS scores were significantly lower in the dextrose group (0.61 ± 0.65, 1.33 ± 0.65, *P *= 0.011). IKDC scores were similar in the dextrose and steroid groups before the procedure (48.34 ± 6.01, 46.66 ± 3.34, *P *= 0.402). In the first week, it was observed to be similar in the dextrose and steroid groups (55.03 ± 8.52, 53.30 ± 1.56, *P *= 0.496) and higher in the dextrose group in the fourth week (62.50 ± 14.46, 53.13 ± 1.60, *P *= 0.036).

Conclusions: It was observed that both dextrose prolotherapy and steroid injections were effective in reducing pain and improving functionality in the treatment of distal SM tendinopathy. Steroid injections showed better results in the first week, while dextrose prolotherapy yielded better outcomes by the fourth week.

## Introduction

Posteromedial knee pain is less common than anterior and lateral knee pain. Medial meniscus tear, Baker cyst, osteoarthritis, and medial collateral ligament sprain are among the causes of posteromedial knee pain. Distal semimembranosus (SM) tendinopathy is also one of the rare causes of posteromedial knee pain [1]. It may occur due to overuse injuries in young athletes, but it is more common in older patients in association with other knee disorders. It is most commonly associated with chondromalacia and degenerative medial meniscus tears [2]. Tendinopathy may develop secondary to adjacent osteophytes in patients with osteoarthritis, and pes anserine bursitis may also accompany tendinopathy [3].

Injuries to coordinated muscles, ligaments, or menisci in the posteromedial aspect of the knee result in anteromedial rotational instability in the knee [4]. The distal SM tendon is important in terms of restricting valgus force in extension, external rotation in flexion, and protecting the posteromedial meniscus from compression in the flexed knee, and it is thought that the diagnosis of tendinopathy is often overlooked [1]. The clinical course of SM tendinopathy may vary, but it usually presents with insidious, progressive pain in the posteromedial knee. The pain increases with activities such as running, cycling, and descending stairs. The pain may radiate to the posteromedial thigh proximal or to the gastrocnemius medial aspect distally. Tenderness is observed at the tendon insertion site on physical examination. Ultrasound (US) and Magnetic resonance imaging (MRI) may be helpful in diagnosis [2]. Segmental thickening, decreased echogenicity and loss of fibrillar echo structure may be observed on US examination. The increase in pain with relatively low pressure of the probe may help distinguish SM tendinopathy from meniscus pathologies that cause pain during physical activity [1].

Although treatment is primarily conservative, steroid injection has a place in resistant cases lasting longer than three months [5]. It is also known that dextrose prolotherapy is effective in tendinopathies [6]. Its effectiveness in distal SM tendinopathy has not been investigated sufficiently. This study aimed to investigate the effectiveness of dextrose prolotherapy in distal SM tendinopathy and compare it with the effectiveness of steroid injection.

## Materials and methods

This retrospective study was conducted by the principles of the Declaration of Helsinki. Informed voluntary consent forms were obtained from the participants. Inclusion criteria were determined as patients between the ages of 18 and 60, having isolated posteromedial knee pain for more than 12 weeks, tenderness at the tendon insertion site on physical examination, tendinosis findings in the distal SM on sonographic examination, and unresponsive to oral medical treatments. Patients with concomitant malignancy, chronic disease, history of inflammatory disease, those with coagulopathy or anticoagulant use, and pregnant women were excluded from the study. Twelve patients who underwent US-guided steroid injection and 13 patients who underwent dextrose prolotherapy treatment with the diagnosis of distal SM tendinopathy between September 01, 2023, and March 28, 2024, at Elazığ Fethi Sekin City Hospital were included in the study.

The procedure was performed after sterile conditions were provided. While the patient was in the prone position, the hamstring tendon was scanned from the proximal with a high-frequency linear US probe and scanned axially until it was attached to the tibia. The distal SM tendon was visualized in the posteromedial aspect of the knee. While the probe was in the transverse position, the in-plane technique was used and the peritendinous area was targeted by entering the probe from the medial side with a 22 G needle at an angle of approximately 45 degrees. After negative aspiration, 2.63 mg Betamethasone sodium phosphate + 5 mg Betamethasone dipropionate + 2 cc prilocaine was injected in the steroid group; 12.5% ​​5 cc dextrose + 2 cc prilocaine was injected in the dextrose group. Isometric knee exercises were recommended to all patients after the injection.

The patient's visual analog scale (VAS) [7] scores before the procedure, one week, and four weeks after the procedure and the International Knee Documentation Committee Subjective Knee Assessment (IKDC) [8] scores were analyzed.

All analyses were performed using SPSS software version 22 (IBM Corp., Armonk, NY). Kolmogorov-Smirnov normality analysis was used to test the data for normal distribution. An independent t-test was used for comparison of independent paired groups. Repeated measures ANOVA test was used for the comparison of more than two dependent groups. A paired sample t-test was used to compare two dependent groups. Continuous variables were presented as mean ± standard deviation and categorical data as percentages. A *P*-value < 0.05 was considered statistically significant.

## Results

Age, body mass index (BMI), and gender distribution of the Dextrose and steroid groups were evaluated. No statistically significant difference was observed between the two groups (Table 1).

**Table 1:** Demographic data of dextrose and steroid groups. The independent t-test was used. BMI, body mass index

	Dextrose group	Steroid group	*P*-value	*f*-value	*t*-value
Gender distribution, female (%)	46	41	0.821	0.570	0.051
Age (years)	40.61 ± 9.13	42.41 ± 8.82	0.621	0.009	0.501
BMI (kg/m^2^)	24.75 ± 2.55	25.30 ± 2.55	0.593	0.004	0.949

The number of patients participating in high-intensity sports or physical activity in the steroid group was five, while it was six in the dextrose group. In the steroid and dextrose group, seven patients had accompanying osteoarthritis. However, there were no patients with synovitis attacks or bursitis.

VAS and IKDC scores of both groups before the procedure, one week after the procedure, and four weeks after the procedure were evaluated. While VAS scores were similar in both groups before the procedure, they were lower in the steroid group in the first week and in the dextrose group in the fourth week. IKDC scores were similar in both groups before the procedure and in the first week, but higher in the dextrose group in the fourth week (Table 2).

**Table 2:** VAS and IKDC scores in the dextrose and steroid groups before the procedure as well as one week and four weeks after the procedure. The independent t-test was used. VAS, visual analog scale; IKDC, International Knee Documentation Committee Subjective Knee Assessment

-	Dextrose group	Steroid group	*P*-value	f-value	*t*-value
Pre-procedure VAS	4.00 ± 0.70	4.33 ± 0.88	0.308	1.730	1.043
First-week VAS	1.84 ± 0.55	1.16 ± 0.93	0.036	9.248	-2.227
Fourth-week VAS	0.61 ± 0.65	1.33 ± 0.65	0.011	0.012	2.755
Pre-procedure IKDC	48.34 ± 6.01	46.66 ± 3.34	0.402	3.918	-0.853
First-week IKDC	55.03 ± 8.52	53.30 ± 1.56	0.496	15.989	-0.692
Fourth-week IKDC	62.50 ± 14.46	53.13 ± 1.60	0.036	38.084	-2.229

Significant changes were observed in VAS and IKDC scores at both follow-up points in both groups (Table 3).

**Table 3:** Temporal change of VAS and IKDC scores in dextrose and steroid groups. *Repeated measures ANOVA test was used. VAS, visual analog scale; IKDC, International Knee Documentation Committee Subjective Knee Assessment

		Pre-procedure	First week	Fourth week	P*(first week/pre-procedure)	P* (Fourth week/pre-procedure)
Dextrose group	VAS	4.00 ± 0.70	1.84 ± 0.55	0.61 ± 0.65	<0.001	<0.001
	IKDC	48.34 ± 6.01	55.03 ± 8.52	62.50 ± 14.46	<0.001	<0.001
Steroid group	VAS	4.33 ± 0.88	1.16 ± 0.93	1.33 ± 0.65	0.002	0.002
	IKDC	46.66 ± 3.34	53.30 ± 1.56	53.13 ± 1.60	0.002	0.002

## Discussion

In our study, it was observed that dextrose prolotherapy and steroid injection were effective on pain and functionality in the treatment of distal SM tendinopathy, and steroid results were better in the first week and dextrose prolotherapy results were better in the fourth week.

The process of connective tissue degeneration and healing after injury is accompanied by neovascularization, increased protein synthesis and matrix changes, hypercellularity, and angiofibroblastic hyperplasia. The mechanical and biochemical properties of fibroblastic scars that change during the process differ from those of native tissues [9]. This approach is effective in treating tendinopathy, fasciopathy, chronic pain, and disabilities resulting from ligament injuries. Therefore, optimizing connective tissue healing. The clinical use of prolotherapy with hypertonic dextrose for optimization has become an available option. The healing cascade, which triggers the inflammatory process and tissue repair, is a key mechanism of prolotherapy and serves as an effective treatment for various musculoskeletal pathologies, including tendinopathies. Steroid replacements are widely used in the control of tendinopathies including distal hamstring tendinopathy due to their anti-inflammatory and analgesic properties [12,13].

Potential advantages of dextrose prolotherapy over steroid injections include the absence of systemic side effects associated with steroid use and the potential for long-term tissue healing [14]. There are also concerns that steroid injections may cause tendon rupture [15]. The main advantage of steroids over prolotherapy is that they provide a more rapid analgesic effect with a strong anti-inflammatory effect. Although this is advantageous in patients who want rapid symptomatic relief, the effects of steroids are generally transient, and the long-term benefits may not be as apparent as those of prolotherapy, and prolotherapy is thought to be more effective in long-term healing [16].

Increasing evidence has shown that prolotherapy is particularly effective in improving functional function [12,17]. In a case series in which dextrose prolotherapy was used to treat chronic spinal pain, most patients experienced significant functional improvement over a follow-up period ranging from two to 30 months [16]. A study of patients with rotator cuff arthropathy showed significantly greater improvements in pain, function, and isometric strength of the shoulder abductor in those receiving dextrose prolotherapy compared with conservative treatment over a one-year follow-up period [12]. Our study results suggest that both dextrose prolotherapy and steroid injection may be effective in the treatment of distal hamstring tendinopathy, but the levels of efficacy vary at different times after injection. While steroid injection provides more rapid pain relief in the short term, dextrose prolotherapy is more effective in the medium term. These findings emphasize the importance of considering the clinical status and preferences of each patient when determining the most appropriate approach to treatment. It can be said that factors such as the patient's pain level, functional goals, and tolerance to possible side effects should be taken into consideration when choosing the treatment method. For example, patients who prioritize a rapid return to sports or physical activity may benefit more from the short-term pain relief provided by steroid injections, while dextrose prolotherapy may be considered the primary option for patients seeking a more permanent solution.

Limitations of this study include the small sample size, short follow-up period, and lack of a control group. Additional research is needed to better understand the long-term outcomes associated with each intervention, the durability of treatment effects, and potential side effects. Larger sample sizes will also allow for more robust statistical analyses and the investigation of potential confounders or effect modifiers.

## Conclusions

It can be concluded that dextrose and steroid injections under US guidance are effective in the treatment of distal SM tendinopathy; steroids are superior for short-term pain relief, while dextrose is more effective for long-term pain management. However, prospective, randomized, long-term clinical studies, including placebo groups, are needed to determine their effectiveness and reliability with more definitive results. Further research, including larger prospective studies, is required to more definitively determine the comparative effectiveness and optimum application of these treatments for distal hamstring tendinopathy.
